# Why Does Advice Discounting Occur? The Combined Roles of Confidence and Trust

**DOI:** 10.3389/fpsyg.2018.02381

**Published:** 2018-11-28

**Authors:** Xiuxin Wang, Xiufang Du

**Affiliations:** ^1^School of Psychology, Shandong Normal University, Jinan, China; ^2^School of Psychology and Cognitive Science, East China Normal University, Shanghai, China

**Keywords:** decision making, advice taking, advice discounting, confidence, trust

## Abstract

Judges tend to discount the opinions of others even though advice is often helpful in improving their accuracy. The present research proposes that this phenomenon of advice discounting results from the judges’ confidence in their initial decision and little trust in advice. Furthermore, the degree of advice discounting may be predicted by the combined roles of confidence and trust. Three studies provide evidence for these hypotheses. Participants were very confident in their initial estimation and had little trust in the advice (study 1). The degree of advice discounting decreased when participants felt less confidence in performing difficult tasks compared with easy tasks (study 2) or when participants placed more trust in advice because the advice was from an expert rather than from a novice (study 3). In addition, confidence and trust predicted the degree of advice discounting across three studies. These findings shed new light on the mechanism underlying advice discounting and advice taking by indicating the combined roles of confidence and trust.

## Introduction

Studies of advice taking have concluded that people tend to overweigh their own opinion relative to advice from an adviser (Harvey and Fischer, 1997; Bonaccio and Dalal, 2006), even though advice often improves judges’ accuracy (Yaniv, 2004b; Gino and Schweitzer, 2008). This phenomenon of advice discounting raises the following significant question: why do people tend to discount others’ advice?

The under-adjustment account asserts that when taking advice, people anchor on their initial judgment and insufficiently adjust away from that anchor (Lim and O’Connor, 1995; Harvey and Fischer, 1997). However, advice discounting still occurs when judges receive advice prior to seeing the decision task (Clement and Krueger, 2000; Rader et al., 2015). In this case, there is no initial decision to serve as an anchor. In fact, people often do not adjust at all, occasionally adjust at an average level, and sometimes fully adjust, showing a tri-modal distribution of adjustment (Soll and Larrick, 2009). This evidence suggests that the under-adjustment account is not an adequate explanation for advice discounting.

An alternative explanation suggests that people have access to their internal justifications for making the initial decision but do not have access to advisers’ reasoning (Yaniv and Kleinberger, 2000; Yaniv, 2004b). Consequently, judges consider their initial estimation to be better supported than advice. However, the discounting effect still exists when people make judgments about novel situations (Cadinu and Rothbart, 1996). In these situations, the amount of supporting evidence for the initial decision and advice is nearly the same. Soll and Mannes (2011) found that people discount advice even when they have no access to supporting evidence, suggesting that the accessibility explanation cannot fully explain the discounting effect.

Confidence in one’s own judgment is a key mechanism underlying advice taking. People are likely to perceive that they are more accurate than advisers are when they feel confident in their judgment (Larrick et al., 2007; Moore and Small, 2007), leading to a lesser need to take advice. Indeed, researchers have found that a higher level of confidence is related to a lower degree of advice taking (See et al., 2011; Gino et al., 2012).

However, evidence from a wide variety of domains has consistently shown that people are highly overconfident (Lichtenstein et al., 1982; Glaser and Weber, 2010). For example, on average, students overestimate their performance on academic exams (Kennedy et al., 2002; Clayson, 2005), and most potential entrepreneurs believe that they have sufficient skills, knowledge and ability to start a business (Koellinger et al., 2007). Similar phenomena exist in the study of human judgment. Lim and O’Connor (1995) explored whether people use model-based forecasts to supplement their own forecast and found that people insistently favor their own estimations over valid models. Gardner and Berry (1995) reported that judges are overconfident in their own judgments relative to advisers’ judgments. Soll and Mannes (2011) found that participants often ignore advice when making a final estimation based on their initial estimation and advice but that they average advice when making decisions based on two pieces of advice, suggesting that participants are very confident in their initial estimation. Considering the significant role of confidence in advice taking, advice discounting may result from judges’ confidence in their initial judgment.

Advice taking is the process by which judges decide how much weight to place on their own judgment and on advice or the process by which judges revise their own opinion based on advice (Sniezek et al., 2004). This perspective suggests that advice taking is influenced not only by judges’ confidence in their own judgment but also by trust in the advice. Confidence alone seems to be insufficient to account for advice discounting. An adequate account should also take the judge’s attitude toward the advice into consideration.

Trust is a significant variable in psychology and is distinguished into two types: affect-based trust and cognition-based trust. Affect-based trust is based on the intentions of others in the sense that the trustee is concerned about the welfare and best interest of the trustor, whereas cognition-based trust is based on performance-relevant cognition, such as ability and expertise (McAllister, 1995). Bonaccio and Dalal (2010) suggested that the adviser’s intentions and expertise are two important factors in determining judges’ weighting of advice. Indeed, both cognition-based trust and affect-based trust play important roles in advice taking (Sniezek and Van Swol, 2001; Gino and Schweitzer, 2008). When people show a high level of trust in advice, they regard the advice as good-quality advice from an adviser with good intentions, and they should place more weight on the advice. However, in previous research, participants were always told that the adviser was an individual who had previously participated in the study (e.g., See et al., 2011; Rader et al., 2015) but were not given further information about the adviser’s level of expertise or integrity. Because the participants did not have sufficient knowledge about the adviser, they may have little trust in the advice. Judges’ trust in advice may play an essential role in advice discounting.

Based on the above, the present research hypothesized that advice discounting may result from judges’ confidence in their own judgment and little trust in the adviser’s advice. Consequently, the degree of advice discounting may be predicted by the combined roles of people’s confidence in their initial decision and trust in advice, while confidence or trust separately may not fully serve as a predictor. We tested these hypotheses in three studies. Study 1 investigated whether people displayed confidence and little trust. Using a task difficulty manipulation, study 2 examined whether advice discounting decreased as judges’ confidence decreased. Study 3 explored whether the discounting effect decreased as expertise-based trust in the adviser’s advice increased. We induced the change in trust by telling the participants that the advice was from an expert or a novice. In addition, we tested whether the degree of advice discounting can be predicted by confidence and trust in all three studies. It is worth noting that, theoretically, both cognition-based trust and affect-based trust play a role in advice discounting. However, for simplicity, we neither measured nor manipulated affect-based trust in our research. We discuss this in more detail in the discussion section.

## Study 1

Study 1 used an estimation task to investigate whether people tend to be very confident in their initial decision and have little trust in advice. Furthermore, study 1 examined whether the degree of advice discounting can be predicted by confidence and trust.

### Methods

#### Participants

Fifty participants (32 females, *M*_age_ = 21.48, *SD*_age_ = 2.10) were recruited in the library of Shandong Normal University. We approached them and asked whether they would like to participate in a study. We offered them a notebook worth approximately ¥5 (nearly $0.8) as compensation for participating. This study was approved by the Human Research Ethics Committee of Shandong Normal University, and written informed consent was obtained from all subjects.

#### Materials and Procedure

Participants sat in private computer cubicles and were asked to complete an estimation task. All of the materials were presented on the computer screen. The participants had an incentive to be accurate; in all studies, we gave the participants an additional notebook if their estimate fell within 10% of the actual number of coins in the photographs.

In phase 1, the participants were shown six photographs of a glass filled with coins and were asked to estimate the number of coins in each photograph. The sequence of the photographs was balanced between subjects using a Latin square design. In total, there were six different sequences. The sequences had no effect on the results in any of the three studies.

In phase 2, the participants were shown the same photographs in the same order, their initial estimation in phase 1 and a piece of advice. The advice was simplified and referred to as “advice from an adviser” without additional information about the adviser. The participants were again asked to estimate the number of coins in each photograph. Importantly, the advice was constant across participants and was determined using the following formula: advice = true value ±20% true value.

Additionally, the participants were asked to complete two questionnaires to measure their confidence in their initial estimation and trust in the advice. Considering that confidence may be affected by advice, half of the participants completed the confidence questionnaire after phase 1, and the other half completed it after phase 2. All participants completed the trust questionnaire after phase 2. The confidence questionnaire was adapted from previous research (See et al., 2011; Gino et al., 2012) and included five items (α = 0.86): “my estimations are remarkably accurate; I am very confident in my own estimates; my estimates are near to the true values; I perform well in this task; how many estimates are in the range of ‘true value -20’ to ‘true value +20’.” The trust questionnaire, adapted from Gino and Schweitzer (2008), also included five items (α = 0.92): “the quality of the advice is really high; the advice is remarkably accurate; the advice is near to the true values; the advisor performs well in this task; how much the advice is in the range of ‘true value -20’ to ‘true value + 20’.” Participants indicated the degree of their agreement from 0 (completely disagree) to 6 (completely agree), or the amounts of estimates and advice that fell in the range from 0 to 6.

### Results

In Table 1, we report the descriptive statistics for the variables that we measured. We used the weight of advice (WOA) to assess the degree of advice taking: WOA = (*final estimation-initial estimation*)/(*advisor’s estimation-initial estimation*). A higher WOA value reflects a greater degree of advice taking. Of the WOAs, 0.67% were undefined values because the initial estimation was equal to the adviser’s estimation. We treated these observations as missing values. Generally, individuals’ final estimates fell between their initial estimates and the advice. However, in a few cases (2.67% of observations), the final estimates fell outside this range such that the WOAs were less than 0 or greater than 1. Following common practice (Soll and Larrick, 2009; See et al., 2011), we truncated these WOAs to the nearer of 0 or 1. The WOAs were closely related (α = 0.85). We used the average of the WOAs to create an index of the extent to which the participant took advice. We also summed and averaged the five confidence items and the five trust items to create the confidence index and the trust index. The confidence did not significantly correlate with the trust, *r* = -0.22, *p* = 0.13.

**Table 1:** Means and SD in studies 1, 2, and 3.

Variable	Error	Distance	WOA	Confidence	Trust
**Study 1**					
*n* = 50	-0.01 (0.80)	-0.01 (0.83)	0.24 (0.22)	3.43 (1.12)	2.22 (1.40)
**Study 2**					
Difficult (*n* = 49)	0.12 (0.75)	0.11 (0.68)	0.45 (0.21)	3.01 (1.23)	3.40 (1.09)
Easy (*n* = 45)	-0.12 (0.48)	-0.12 (0.40)	0.36 (0.19)	3.52 (1.07)	3.37 (0.99)
**Study 3**					
Expert (*n* = 51)	0.01 (0.58)	-0.47 (0.44)	0.51 (0.20)	3.22 (1.07)	3.88 (0.73)
Novice (*n* = 53)	-0.01 (0.63)	0.45 (0.70)	0.20 (0.19)	3.01 (1.25)	1.75 (1.11)

*The error (in initial estimates) was calculated by averaging the standardized absolute errors across six trials. Similarly, the distance (between advice and the initial estimate) was calculated by averaging the standardized absolute distance across six trials*.

#### The Phenomenon of Advice Discounting

The mean WOA was 0.24. This value is similar to values that have been reported in previous research (Harvey and Fischer, 1997; Soll and Larrick, 2009) and demonstrates that the phenomenon of advice discounting exists.

#### The Existence of Overconfidence and Little Trust

Participants’ errors of initial estimates in each trial, calculated by subtracting the true values from the initial estimates, were -8.06 (3.29), -57.7 (19.83), -89.78 (31.86), -112.74 (49.91), -146.18 (66.28), and -176.84 (154.33). Participants’ errors of final estimates in each trial, calculated by subtracting the true values from the final estimates, were -6.68 (3.97), -51.08 (18.27), -79.48 (29.28), -78.22 (64.39), -95 (89.41), and -112.92 (123.98). On average, the participants underestimated the number of coins. The mean confidence was 3.43, suggesting that people were very confident in their initial decision. The mean value for trust was 2.22, indicating that people had little trust in the adviser’s estimation.

#### Confidence and Trust Predicted Advice Discounting

No order effect was found for participants who answered the confidence questions after phase 1 or after phase 2, *t*(48) = 1.00, *p* = 0.33, 95% CI [-0.32, 0.95], *d* = 0.32. The correlation between confidence after phase 2 and distance between advice and the initial estimate was not significant (*r* = 0.06, *p* = 0.78), suggesting that a larger distance did not make people less confident. Thus, we report the results collapsed across the order conditions. A multiple regression analysis showed that confidence and trust predicted WOA, *R^2^* = 0.51, *F*(2, 47) = 24.50, *p* < 0.001; *b*_confidence_ = -0.07, *t* = -3.51, *p* < 0.01; *b*_trust_ = 0.08, *t* = 5.14, *p* < 0.001.

## Study 2

Study 2 explored whether advice discounting decreased as confidence in the initial decision decreased and whether confidence and trust predicted advice discounting. Participants should be less confident in their initial estimation when confronted with difficulty because they have less information to use in making their estimation. Thus, we used a difficult/easy task to manipulate confidence. Specifically, study 2 investigated whether advice discounting decreased in difficult tasks compared to easy tasks.

### Methods

#### Participants

Ninety-four participants were recruited to participate in the study through the same method employed in study 1 (67 females, *M*_age_ = 23.02, *SD*_age_ = 2.51). As in study 1, we offered them a notebook as compensation for participating.

#### Design and Procedure

We asked the participants to engage in an estimation task and randomly assigned them to one of the two task difficulty conditions. In difficult tasks, the six photographs of a glass filled with coins were blurred (Gino and Moore, 2007). By contrast, in easy tasks, the pictures were clear.

The participants first completed two practice trials with feedback on the actual number. Then, they estimated the number of coins in each photograph. After that, they were asked to complete the confidence questionnaire (α = 0.89). They were shown advice and were asked to re-estimate the number of coins. Finally, they completed the trust questionnaire (α = 0.88).

### Results

Of the WOAs, 0.33% were missing because the initial estimations or final estimations were unrecorded. Furthermore, 3.46% of the WOAs were regarded as missing data because the initial estimation was equal to the adviser’s estimation. In addition, 3.96% of the observations were less than 0 or greater than 1 and were truncated to the nearer of 0 or 1. Similar to study 1, in study 2, the WOAs showed high internal consistency (α = 0.79) and were averaged to create an index of advice taking.

#### Manipulation Check

The accuracy of the initial estimation in easy tasks tended to be greater than that in difficult tasks, *t*(92) = -1.86, *p* = 0.07, 95% CI [-0.51, 0.02], *d* = 0.38. Participants in the easy condition felt more confidence in their initial estimation than did participants in the difficult condition, *t*(92) = 2.13, *p* = 0.04, 95% CI [0.04, 0.99], *d* = 0.44; however, these two groups showed no difference in trust, *t*(92) = -0.17, *p* = 0.87, 95% CI [-0.46, 0.39], *d* = 0.03 (for average scores, please see Table 1). In addition, the confidence significantly correlated with the trust, *r* = 0.23, *p* < 0.05. These results showed that the task difficulty manipulation was effective.

#### The Effect of Task Difficulty on Advice Taking

The distance between advice and the initial estimate was calculated by averaging the standardized absolute distance across six trials (α = 0.88). An independent-samples *t-*test was used for statistical analysis and showed that the distance in difficult tasks tended to be significantly farther than that in easy tasks (Table 1), *t*(92) = 1.98, *p* = 0.05, 95% CI [0.00, 0.46], *d* = 0.41. Considering that this distance could affect advice taking (Yaniv, 2004a), we treated it as a covariate in the following analysis. Notably, all effects remained significant when the covariate was not included. The covariance analysis showed that participants weighted advice more heavily in the difficult tasks than in the easy tasks, *F*(1, 91) = 3.97, *p* < 0.05, ${\text{η}}_{\text{p}}^{2}$ = 0.04. The main effect of distance was not significant, *F*(1, 91) = 0.49, *p* = 0.49, ${\text{η}}_{\text{p}}^{2}$ = 0.01.

#### Confidence and Trust Predicted Advice Discounting

To examine the role of confidence and trust in advice discounting, we performed a multiple mediation analysis with confidence and trust as parallel mediators. We conducted this analysis with model 4 of the process procedure (Hayes, 2013) using 5000 bootstrap iterations. We included the distance between advice and the initial estimate as a covariate. Notably, all effects remained the same when the covariate was not included. Together, confidence and trust did not mediate the effect of task difficulty on advice taking, 95% CI [-0.00, 0.08]. When considering the specific indirect effect of the two mediators separately, the indirect effect of confidence was significant, 95% CI [0.00, 0.07], whereas the indirect effect of trust was not, 95% CI [-0.03, 0.05].

In addition, Model 1 of the mediation analysis (i.e., the effect of task difficulty on confidence) showed that task difficulty significantly predicted confidence, *b* = -0.50, *t* = -2.02, *p* < 0.05, 95% CI [-0.99, -0.01]. Model 2 of the mediation analysis (i.e., the effect of task difficulty on trust) showed that task difficulty did not significantly predict trust, *b* = 0.11, *t* = 0.52, *p* = 0.60, 95% CI [-0.32, 0.55]. These results were consistent with the results of manipulation checks. More importantly, Model 3 of mediation analysis (i.e., the effect of task difficulty, confidence and trust on WOA) showed that confidence and trust significantly predicted WOA, *b*_confidence_ = -0.05, *t* = -2.99, *p* < 0.01, 95% CI [-0.08, -0.02]; *b*_trust_ = 0.08, *t* = 4.33, *p* < 0.001, 95% CI [0.05, 0.12]; *b*_cifficulty_ = 0.06, *t* = 1.54, *p* = 0.13, 95% CI [-0.02, 0.14].

## Study 3

Study 3 was designed to explore whether advice discounting decreased as cognition-based trust in advice increased. When advice is received from an expert rather than from a novice, participants should place more trust in the advice. Consequently, advice discounting should decrease when the advice is from an expert. Furthermore, whether confidence and trust predicted advice discounting.

### Methods

#### Participants

One hundred and four participants participated in the study (62 females, *M*_age_ = 21.01, *SD*_age_ = 1.55). As in study 1, we offered them a notebook as compensation for participating.

#### Design and Procedure

Participants were asked to engage in an estimation task and were randomly assigned to one of two conditions. As in study 2, the participants first completed two practice trials with feedback on the actual number. Then, they estimated the number of coins in each photograph. After that, they were asked to complete the confidence questionnaire (α = 0.83). Finally, they were shown advice and were asked to re-estimate the number of coins. We told the participants that the adviser was a participant who had previously participated in a similar study. In the expert condition, we also told the participants that the adviser was trained on and capable of performing this task. We told the participants in the novice condition that the adviser was not trained and that the accuracy of the adviser’s estimation was average. Notably, the advice from the expert was more accurate than the advice from the novice. Finally, the participants completed the trust questionnaire (α = 0.93).

We determined the advice by a pilot study. We recruited one hundred participants who were not involved in any of the main experiments. Fifty participants estimated six different photographs for practice before estimating the six photographs used in the main experiment. In the practice trials, there was feedback on the actual number. The other 50 participants did not have a practice session. The accuracy of estimations from participants with practice was significantly higher than that from participants without practice (*ps* < 0.001). We used the mean estimations from the participants with practice as the advice of the expert, whereas we used the mean estimations from participants without practice as the advice of the novice.

### Results

Of the WOAs, 4.64% were treated as missing values because the initial estimation was equal to the adviser’s estimation. Furthermore, 4.17% of observations were less than 0 or greater than 1 and were truncated to the nearer of 0 or 1. The WOAs were closely related (α = 0.82) and were averaged to create an overall measure of WOA.

#### Manipulation Check

Participants who received expert advice reported more trust in the advice than did participants who received novice advice, *t*(102) = -11.48, *p* < 0.001, 95% CI [-2.50, -1.76], *d* = 2.27. However, confidence in the initial decision did not significantly differ between these two conditions, *t*(102) = -0.88, *p* = 0.38, 95% CI [-0.65, 0.25], *d* = 0.18 (average scores of the confidence and trust scales are given in Table 1). In addition, the confidence did not significantly correlate with the trust, *r* = 0.15, *p* = 0.13. These results showed that the manipulation of adviser’s expertise worked.

#### The Effect of the Adviser’s Expertise on Advice Taking

The distance between advice and the initial estimate in the expert condition was significantly nearer than that in the novice condition, *t*(102) = 8.01, *p* < 0.001, 95% CI [0.69, 1.15], *d* = 1.57 (for average scores, please see Table 1). This is because the initial estimates were quite good in both conditions, and the advice was better in the expert condition. Thus, we treated it as a covariate in the following analysis. Notably, all effects remained significant when the covariate was not included. An analysis of covariance found that the participants weighted advice more heavily in the expert condition than in the novice condition, *F*(1, 101) = 40.64, *p* < 0.001, ${\text{η}}_{\text{p}}^{2}$ = 0.29. The main effect of distance was not significant, *F*(1, 101) = 0.00, *p* = 0.99, ${\text{η}}_{\text{p}}^{2}$ = 0.00.

#### Confidence and Trust Predicted Advice Discounting

As in study 2, we performed a multiple mediation analysis with confidence and trust as parallel mediators to examine the role of confidence and trust in advice discounting. We included the distance between advice and the initial estimate as a covariate. Notably, all effects remained the same when the covariate was not included. Together, confidence and trust mediated the effect of adviser’s expertise on WOA, 95% CI [0.06, 0.22]. When considering the specific indirect effect of the two mediators separately, the indirect effect of the trust was significant, 95% CI [0.07, 0.23], whereas the indirect effect of confidence was not, 95% CI [-0.04, 0.02].

In addition, Model 1 of the mediation analysis (i.e., the effect of adviser’s expertise on confidence) showed that adviser’s expertise did not significantly predict confidence, *b* = 0.13, *t* = 0.46, *p* = 0.65, 95% CI [-0.44, 0.70]. Model 2 of the mediation analysis (i.e., the effect of adviser’s expertise on trust) showed that adviser’s expertise significantly predicted trust, *b* = 1.75, *t* = 7.50, *p* < 0.001, 95% CI [1.29, 2.21]. These results were consistent with the results of manipulation checks. More importantly, Model 3 of mediation analysis (i.e., the effect of adviser’s expertise, confidence and trust on WOA) showed that confidence and trust significantly predicted WOA, *b*_confidence_ = -0.05, *t* = -3.23, *p* < 0.01, 95% CI [-0.08, -0.02]; *b*_trust_ = 0.08, *t* = 4.28, *p* < 0.001, 95% CI [0.04, 0.12]; *b*_expertise_ = 0.15, *t* = 2.72, *p* < 0.01, 95% CI [0.04, 0.26].

## General Discussion

Across three studies, the present research provided evidence for an alternative explanation for advice taking: the combined effect of confidence and trust. Specifically, in study 1, people discounted advice and displayed confidence in their initial estimation and little trust in the adviser’s advice. Study 2 showed that advice discounting decreased when confidence decreased. Study 3 found that people were more receptive to advice when trust increased. Furthermore, the degree of advice taking or advice discounting can be predicted by confidence and trust.

Advice discounting occurs when individuals underweight an advice that is better than their initial judgment. However, how can we talk about advice discounting when participants may have no idea about the accuracy of the advice in relation to their own? Individuals’ estimates can be expressed as the sum of the true value and random error (Yaniv and Kleinberger, 2000). This statistical view suggests that averaging initial estimates and the advice improves accuracy of the final estimate. In other words, averaging is often the optimal strategy when individual combines their initial estimate and the advice (Yaniv and Kleinberger, 2000; Soll and Larrick, 2009). In the term of WOA, advice discounting occurs when the WOA is less than 0.5. In our study 1, the WOA was 0.24, which suggested a significant phenomenon of advice discounting. It is also the case in study 3. However, differential weighting of opinions may be warranted when additional information is available. For instance, when people know that the advice is good, they should overweight the advice, and the WOA should be significantly larger than 0.5. However, though participants assigned to expert condition in our study 2 were informed that the advice was from an expert, the WOA was just 0.51. In general, participants in our studies generally discounted advice.

The phenomenon of advice discounting has received considerable attention from researchers (Harvey and Fischer, 1997; Bonaccio and Dalal, 2006). The under-adjustment account (Lim and O’Connor, 1995) and accessibility explanation (Yaniv and Kleinberger, 2000) have been proposed as mechanisms underlying this phenomenon, but evidence suggests that they cannot fully account for it (Soll and Larrick, 2009; Soll and Mannes, 2011). The present research proposed and proved an alternative explanation for advice discounting: the combined roles of confidence and trust.

Advice taking involves the process by which judges revise their own opinion based on advice. Confidence in one’s initial judgment and trust in advice should both play important roles in advice taking. Evidence supports this proposal. For instance, See et al. (2011) suggested that people with high power tend to discount advice more than people with low power do and that confidence mediates this relationship. Gino et al. (2012) found that people who have higher levels of anxiety tend to be less confident and accept more advice from advisers than do those with lower levels of anxiety. Gino and Schweitzer (2008) demonstrated that trust mediated the relationship between the emotions of gratitude/anger and advice taking.

However, people are, on average, very confident and show little trust. As one of the mainstays of heuristics and biases in behavioral decision-making, overconfidence biases exist in many domains (see, for example, Kahneman, 2011, Chapter 22) and across different cultures (Stankov and Lee, 2014). In previous research, participants may have little trust in the adviser because they had little information about the adviser. In reality, judges are often familiar with the adviser, which explains why some people are sometimes receptive to others’ advice in real life. People may discount advice because they are overly confident in their initial decision and have little trust in the advice.

The confidence and little trust explanation for advice discounting is quite different from the under-adjustment account and accessibility explanation. The under-adjustment account asserts that advice discounting results from judges’ insufficient adjustment from the anchor (Harvey and Fischer, 1997), which mainly involves the underlying cognitive process of advice taking. The accessibility explanation contends that different levels of accessibility to the reasoning of judges or advisers account for advice discounting (Yaniv, 2004b). This explanation mainly involves fewer justifications of the advice. Unlike the above two explanations, the confidence and little trust explanation of advice discounting takes into consideration judges’ attitudes toward both their initial decision and the advice. However, it is difficult to conclude that these three explanations are completely unrelated. Perhaps judges’ attitudes toward their initial decision and the advice are based on the accessibility of the reasoning of the initial decision and advice. More research is needed to understand the relationship between these three interpretations of advice discounting.

Notably, our manuscript mainly focused only advice discounting. Yet, advice discounting varies according to several factors. Furthermore, there are also situations in which people overweight advice. For instance, Gino (2008) suggested that individuals overweighed advice from others when the advice cost money. An overweighting may also occur due to social reasons: one may want to be polite, there may be social pressure, and one does not want to hurt someone and so on. Researchers may further examine under what conditions people discount advice and under what conditions people overweight advice.

It also should be note that we focused on situations in which people receive unsolicited advice. However, there are also situations in which people ask for advice. In situations people ask for advice, people may behave less advice discounting because of the paid effect we mentioned above (Gino, 2008). In addition, people may have no initial decisions, or have little confidence in their initial decisions. Therefore, people may place relative more trust in advice. Considering these distinctions between these two situations, we are cautious to conclude that our findings could be generalized to the situations in which people ask for advice.

In addition to proposing and showing the confidence and little trust account of advice discounting, the present research contributes to the area of advice taking by suggesting the combined roles of confidence and trust mechanism. In all three studies, results showed that advice taking could be predicted by confidence and trust. Compared to the confidence and trust separately, the combined roles of confidence and trust has some advantages. Firstly, factors that have an effect on advice taking may not only affect confidence, but also affect trust. For instance, power could elevate individuals’ confidence (See et al., 2011), and may undermine trust (Inesi et al., 2012). In this way, confidence and trust separately could not fully account for the effect of power on advice taking. Secondly, confidence mainly concerns individuals’ attitudes toward their initial estimations. While trust mainly concerns individuals’ attitudes toward the advice. An adequate account of advice taking should take into consideration the judge’s attitude not only toward their initial decision but also toward the advice. The combined roles of confidence and trust does so, and this makes it be a better predictor of advice taking.

Some limitations of this study should be mentioned. First, affect-based trust also plays a role in advice discounting (e.g., see Mackinger et al., 2017). However, for simplicity, we neither measured nor manipulated affect-based trust in our research. Mackinger et al. (2017) found that bank customers indicated less cooperation with their advisors when they were uncertain about the advisors’ intention. However, there was a conflict of interest between bank customers and their advisors in their study. This may not be the case in our study. The advice given to the participants in studies 1 and 3 was simplified and referred to as “advice from an adviser” without additional information about the adviser. Our informal survey after the study suggested that most participants believed that the adviser was the experimenter. Our participants may not have doubted the intention of the adviser considering that there was no conflict of interest. Admittedly, other possibilities exist. We cannot ensure that affect-based trust was not involved in advice taking or that the unclear description did not affect advice taking. Future research could include affect-based trust to explain advice discounting or advice taking.

Second, we manipulated not only the perceived expertise but also the actual advice. Given that participants might perceive the accuracy of the advice, manipulating only the perceived expertise seemed to be insufficient. On average, expert advice is better than advice from a novice. However, this introduced noise in that the advice varied between conditions. Perhaps the expert effect is because that the advice is better.

## Conclusion

Advice taking involves the process by which judges revise their own opinion based on advice. Judges’ confidence in their initial decision and their trust in advice both play significant roles in advice taking or advice discounting. Extending previous research, we proposed and showed that advice discounting results from the judges’ confidence in their initial decision and little trust in advice, and that the degree of advice discounting or advice taking can be predicted by the combined roles of confidence and trust.

## Author Contributions

XW contributed to the initial idea conception, collected, analyzed, and interpreted the data, and wrote this manuscript. XD was responsible for supervision (initial idea and experimental design improvements), interpretation of the results, and a critical review of the manuscript.

## Conflict of Interest Statement

The authors declare that the research was conducted in the absence of any commercial or financial relationships that could be construed as a potential conflict of interest.
